# ChoK-Full of Potential: Choline Kinase in B Cell and T Cell Malignancies

**DOI:** 10.3390/pharmaceutics13060911

**Published:** 2021-06-20

**Authors:** Samantha Gokhale, Ping Xie

**Affiliations:** 1 Department of Cell Biology and Neuroscience, Rutgers University, 604 Allison Road, Nelson Labs Room B336, Piscataway, NJ 08854, USA; sg1228@scarletmail.rutgers.edu; 2 Graduate Program in Cellular and Molecular Pharmacology, Rutgers University, Piscataway, NJ 08854, USA; 3 Rutgers Cancer Institute of New Jersey, New Brunswick, NJ 08903, USA

**Keywords:** choline metabolism, choline kinase, B cell malignancies, T cell lymphomas, TRAF3, cancer therapy

## Abstract

Aberrant choline metabolism, characterized by an increase in total choline-containing compounds, phosphocholine and phosphatidylcholine (PC), is a metabolic hallmark of carcinogenesis and tumor progression. This aberration arises from alterations in metabolic enzymes that control PC biosynthesis and catabolism. Among these enzymes, choline kinase α (CHKα) exhibits the most frequent alterations and is commonly overexpressed in human cancers. CHKα catalyzes the phosphorylation of choline to generate phosphocholine, the first step in de novo PC biosynthesis. CHKα overexpression is associated with the malignant phenotype, metastatic capability and drug resistance in human cancers, and thus has been recognized as a robust biomarker and therapeutic target of cancer. Of clinical importance, increased choline metabolism and CHKα activity can be detected by non-invasive magnetic resonance spectroscopy (MRS) or positron emission tomography/computed tomography (PET/CT) imaging with radiolabeled choline analogs for diagnosis and treatment monitoring of cancer patients. Both choline-based MRS and PET/CT imaging have also been clinically applied for lymphoid malignancies, including non-Hodgkin lymphoma, multiple myeloma and central nervous system lymphoma. However, information on how choline kinase is dysregulated in lymphoid malignancies is very limited and has just begun to be unraveled. In this review, we provide an overview of the current understanding of choline kinase in B cell and T cell malignancies with the goal of promoting future investigation in this area.

## 1. Introduction

Aberrant choline metabolism, characterized by an increase in total choline-containing compounds (tCho), phosphocholine (P-Cho) and phosphatidylcholine (PC), is a metabolic hallmark of carcinogenesis and tumor progression [1,2,3,4,5,6]. Phosphatidylcholine is the most abundant phospholipid of all mammalian cell types and subcellular organelles, comprising ~50% of total cellular phospholipids [7,8]. All cancers tested so far display abnormal PC metabolism, which has been detected with non-invasive magnetic resonance spectroscopy (MRS) approaches in cancer patients and animal models of cancers [1,2,3,4]. Therefore, MRS-based measurement of choline metabolism has been useful in clinical diagnosis and treatment monitoring of cancer patients [1,2,3,4]. Mechanistic investigation has elucidated that the molecular causes of aberrant choline metabolism in cancers arise from alterations in enzymes that control the anabolic and catabolic pathways of PC, including choline kinase α (CHKα), PC-specific phospholipase C (PC-PLC), PC-PLD1 and glycerophosphocholine phosphodiesterases (GPCPDs), which are often associated with up-regulated expression and activity of several choline transporters [1,2,4,6]. Thus, these metabolic enzymes and choline transporters have been identified as important therapeutic targets for anti-cancer therapy, either alone or in combination with other cancer therapies [1,2,4,6].

Among all the enzymes that control choline metabolism, CHKα exhibits the most frequent alterations in human cancers [1,3,6,9]. Choline kinase is a cytosolic enzyme that catalyzes the phosphorylation of choline to generate P-Cho in the presence of ATP and magnesium, the first step in de novo PC biosynthesis termed the Kennedy pathway [9,10,11]. This enzyme exists in mammalian cells as three isoforms, CHKα1, CHKα2 and CHKβ, which form active hetero- or homo-dimers and are encoded by two separate genes, *CHKA* and *CHKB* in human [9,10,12,13]. CHKα1 and CHKα2 isoforms are generated by alternative splicing of the *CHKA* gene and share the same catalytic activity [10,13]. CHKα and CHKβ display differential tissue expression patterns [9,12,13]. *Chka*^−/−^ mice die early in embryogenesis, while *Chkb*^−/−^ mice survive to adulthood but develop hindlimb muscular dystrophy and forelimb bone deformity, suggesting a broader function for Chkα than Chkβ [9,12,13]. In line with this, CHKα overexpression has been detected in a wide variety of human cancers, including breast, prostate, lung, colon, liver, head and neck, esophageal, stomach, bladder, ovary, skin and brain cancers, with an incidence of 40–60% in all tumors investigated [3,6,9]. Major mechanisms leading to CHKα overexpression include amplification of the *CHKA* gene, activation of oncogenic signaling pathways and epigenetic alterations (such as HDACs) [1,9,11,14,15,16,17]. Examples of oncogenic pathways that induce the up-regulation of CHKα are Ras-Raf-MAPK-AP1, PI3K-mTOR-Akt, EGFR-PI3K-Akt, Bcr-Abl-MEK-ERK/PI3K-Akt, and c-Kit-Ras-Raf-MAPK/PI3K-Akt [1,9,11,16]. CHKα overexpression is associated with the malignant phenotype, metastatic capability and drug resistance in human cancers, and thus has been recognized as a robust biomarker in cancer [3,6,11,18]. Interestingly, therapeutic targeting of CHKα in cancer models has revealed a complex reciprocal interaction between oncogenic signaling pathways and choline metabolism [1,3,15]. For example, inhibition of choline metabolism by CHKα inhibitors or genetic silencing of CHKα by siRNA results in reduced PI3K-mTOR-Akt, Ras-Raf-MAPK-AP1 or EGFR-mTorc2 signaling in different cancer models, highlighting a causal role of CHKα elevation in carcinogenesis and tumor progression [1,3,15]. CHKα has consequently been identified as a promising drug target for cancer therapy [1,3,6].

The identification of CHKα as a potential therapeutic target for human cancers has attracted interests in developing pharmacological inhibitors of this enzyme. A variety of CHKα inhibitors have been synthesized or developed by means of various approaches, including molecular modifications of the prototype CHKα inhibitor hemicholinium-3 (HC-3), computer-based drug design and screening of natural products [3,6]. Chemical modifications of HC-3 have resulted in the synthesis of two lead compounds of CHKα inhibitors in Dr. Juan Carlos Lacal’s laboratory, MN58b and RSM932A (also known as TCD-717), which represent the structures of bis-pyridinium and bis-quinolinium compounds, respectively [3,6]. Additional modifications of MN58b and RSM932A generated novel potent CHKα inhibitors represented by EB-3D and EB-3P, respectively [19,20]. The use of the CHKα structure for in silico screening has led to the discovery of next-generation CHKα inhibitors represented by CK37, a non-symmetric compound [3,6]. Other next-generation CHKα inhibitors (such as V-11-023907 and V-11-0711, two non-symmetric inhibitors) were developed using a compound library screening approach [3,6]. Detailed structures of these representative CHKα inhibitors are illustrated in two excellent reviews [3,6]. In general, CHKα inhibitors suppress the choline kinase activity of this enzyme and reduce PC biosynthesis, leading to decreased P-Cho and PC levels in cells [1,3,6]. Many CHKα inhibitors such as MN58b, RSM932A, EB-3D and CK37 exhibit potent anti-proliferative effects on cancer cells through induction of apoptosis in vitro and are able to retard tumor growth in mouse xenograft models of human cancers in vivo [1,3,6,20].

Of clinical importance, increased expression and activity of CHKα and choline transporters have also spurred the successful development of radiolabeled choline analogs, [^11^C]-choline or [^18^F]-fluorocholine, as tracers for positron emission tomography/computed tomography (PET/CT) in diagnostic imaging of cancer patients for detection, staging and monitoring response to therapies [2,5]. Radiolabeled choline tracers are particularly useful for diagnostic imaging of brain tumors, prostate cancer, lung cancer, esophageal cancer and hepatocellular carcinoma, in which the commonly used glucose tracer [^18^F]-2-fluoro-2-deoxyglucose (FDG) lacks specificity or sensitivity [2,5]. Interestingly, both MRS imaging of tCho and PET/CT imaging with [^11^C]-choline or [^18^F]-fluorocholine tracers have also been clinically applied for the diagnosis of lymphoid malignancies, including non-Hodgkin lymphoma (NHL), multiple myeloma (MM) and central nervous system lymphoma (CNSL) [21,22,23,24,25,26,27,28,29,30,31]. However, in contrast to the well-documented oncogenic alterations and pathways that cause CHKα overexpression in solid tumors, information on how choline kinase is dysregulated in lymphoid malignancies is very limited and has just begun to be unraveled. On the other hand, it is known that the de novo biosynthesis of PC is critical for the proliferation, activation, germinal center (GC) reaction, immunoglobulin (Ig) isotype switching and antibody production of normal B lymphocytes or plasma cells [32,33,34,35]. In this context, here we provide an overview of the current understanding of choline kinase in B cell and T cell malignancies with the goal of promoting future investigation in this area.

## 2. Genetic Alterations of Choline Kinase in Human Lymphoid Malignancies

**Figure 1:**
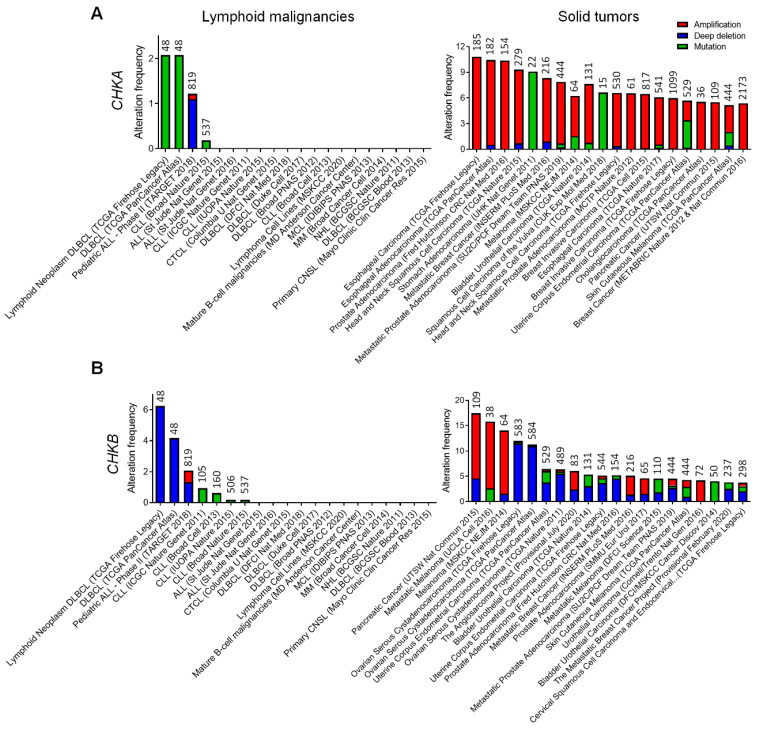
Genetic alterations of choline kinase in lymphoid malignancies versus solid tumors. Representative results of *CHKA* (**A**) and *CHKB* (**B**) are retrieved from TCGA. All the datasets of lymphoid malignancies available at TCGA are shown, while twenty datasets of solid tumors that exhibit a relatively higher frequency of genetic alterations are selected and presented in the graphs. Genetic alterations shown include amplification (copy number gain), deep deletion (copy number loss) and mutation (including missense mutation, frameshift insertion or deletion, and in-frame insertion or deletion). The sample size of each dataset is indicated on top of each bar in the graphs.

To obtain information on genetic alterations of choline kinase in human cancers, we searched the Cancer Genome Atlas (TCGA) [36] for the presence and frequency of gene amplification, deletion and mutation on *CHKA* and *CHKB* in various human lymphoid malignancies as compared to solid tumors. We found that genetic alterations of *CHKA* occur at a very low frequency (< 2.3%) in diffuse large B cell lymphoma (DLBCL), pediatric acute lymphoid leukemia (ALL) and chronic lymphocytic leukemia (CLL) and are absent in all other lymphoid malignancies (Figure 1A). In contrast, gene amplification of *CHKA* is a recurrent genetic alteration and contributes to CHKα overexpression in a variety of solid tumors, including esophageal, liver, prostate, head and neck, stomach, breast, skin, bladder, uterine and pancreatic cancers [14,15] (Figure 1A). Interestingly, gene amplification of *CHKB* is only detected in pancreatic cancer and melanoma, while gene deletion of *CHKB* is documented in ovary, uterine and several other solid tumors (Figure 1B). Gene deletion of *CHKB* is also detected in DLBCL (~4–6%) and pediatric ALL (~1%), while amplification and mutation of *CHKB* are very rare in human lymphoid malignancies (Figure 1B). Thus, a lack of *CHKA* and *CHKB* gene amplification in lymphoid malignancies suggests that aberrant choline metabolism in these diseases is mediated by genetic mechanisms at least partially distinct from those discovered in solid tumors.

Consistent with their pattern of genetic alterations observed in human cancers, overexpression of CHKα is oncogenic in human embryonic kidney 293T (HEK293T) or Madin-Darby canine kidney (MDCK) cells, while CHKβ overexpression is not sufficient to induce cell transformation nor in vivo tumor growth [9,12,13]. On the other hand, recessive loss-of-function mutations in *CHKB* lead to megaconial congenital muscular dystrophy in patients, which resembles the hindlimb muscular dystrophy observed in *Chkb*^−/−^ mice and suggests a role for CHKβ in regulating mitochondrial physiology in muscle fibers [9,12,13,37]. However, the functional consequence and significance of *CHKB* deletion in human lymphoid malignancies and solid tumors remain unclear. In this regard, Gruber et al. reported in vitro evidence suggesting that the balance of the α and β isoforms of CHK is critical for cancer cell survival [38]. It is conceivable that *CHKB* deletion would result in an altered balance of the α versus β isoforms and an increased ratio of CHKα in cancer cells. Whether an increased ratio of CHKα resulting from *CHKB* deletion plays an oncogenic role in lymphoid malignancies requires further investigation.

## 3. Elevated CHKα Expression and Choline Metabolism in B Cell Malignancies

Initial MRS imaging studies revealed that abnormal choline metabolism is detectable in patients and animal models with NHL and CNSL as demonstrated by the increased choline to creatine (Cho/Cr) and choline to water (Cho/water) ratios in the lymphomas, indicating that MRS imaging of choline metabolism is helpful for both diagnosis and post-treatment surveillance [21,22,23,24,25,26,39,40,41,42,43]. More recently, PET/CT imaging studies with [^11^C]-choline or [^18^F]-fluorocholine have allowed the detection of DLBCL and Hodgkin lymphoma (HL) in patients with recurrent prostate cancer [28,44,45]. PET/CT imaging with [^11^C]-choline or [^18^F]-fluorocholine has actually become a useful tool for staging and assessment of therapeutic response in patients with NHL, MM and CNSL, and has demonstrated much higher sensitivity and specificity for MM and CNSL than the widely used [^18^F]-FDG [27,29,30,31]. However, the specificity of choline tracers is not absolute, as [^11^C]-choline or [^18^F]-fluorocholine is also accumulated in tissues with sterile inflammation and bacterial/viral infections [2,5,46]. Overall, when used alongside traditional [^18^F]-FDG PET/CT imaging, [^11^C]-choline or [^18^F]-fluorocholine PET/CT can improve diagnostic accuracy and sensitivity of B cell malignancies for early detection and prognostic stratification as well as detection of minimal residual disease (MRD) after treatment.

Complementary to the in vivo imaging evidence, metabolomic analyses of serum or plasma samples of patients and animal models with B cell malignancies consistently reveal evidence of choline metabolic alterations, including decreased serum or plasma levels of choline, PC, lysophosphatidylcholine (LPC) and glycerophosphocholine (GPC) due to excessive choline uptake by malignant B cells [47,48,49,50]. These metabolomic data, together with the evidence from choline-based PET/CT imaging, indicate that increased choline uptake and elevated choline kinase activity are present in NHL, MM and CNSL. However, information on choline transporter or CHKα overexpression in B cell malignancies is very sparse in the published literature. We thus surveyed the public gene expression databases of human cancers at Oncomine (http://www.oncomine.org (accessed on 26 April 2021 and 1 June 2021)) for the expression levels of choline transporter genes [51], *CHKA* and *CHKB* in human B cell malignancies in comparison to normal B lymphocytes. According to the datasets available at Oncomine, no significant changes in the expression of *SLC5A7* (encoding choline transporter 1, CHT1), *SLC22A4* (encoding carnitine/organic cation transporter 1, OCTN1) and *SLC22A5* (encoding OCTN2) are detected in human B cell malignancies. Interestingly, the expression of *SLC44A1* (encoding choline transporter-like protein 1, CTL1) is up-regulated in human DLBCL and HL [52,53,54]; the expression of *SLC22A3* (encoding organic cation transporter 3, OCT3) is up-regulated in follicular lymphoma (FL) and HL [52,54]; and *CHKA* expression is significantly increased in human DLBCL and Burkitt’s lymphoma (BL) [55,56,57,58]. In contrast, the expression of both *SLC22A1* (encoding OCT1) and *SLC22A2* (encoding OCT2) is down-regulated in human centroblastic lymphoma (CL) and DLBCL [52,58], while *CHKB* expression is significantly decreased in human BL and HL [53,54,58]. Decreased *CHKB* expression would likely result in an increased ratio of CHKα in malignant B cells. The above expression profile suggests that up-regulation of *CHKA*, but not *CHKB*, may contribute to the increased choline kinase activity in B cell malignancies. However, given the lack of *CHKA* gene amplification, it remained unknown what oncogenic alterations and signaling pathways cause the elevated expression of *CHKA* in B cell malignancies. Addressing this gap in knowledge, we recently made an interesting finding that deletion of the tumor suppressor gene *TRAF3* leads to up-regulation of *Chka* at mRNA and protein levels in both mouse and human models of B cell malignancies [59].

TRAF3 is a cytoplasmic adaptor protein and an E3 ubiquitin ligase that regulates the signal transduction pathways of a diverse array of immune receptors, including the tumor necrosis factor receptor (TNF-R) superfamily, pattern recognition receptors (PRRs) and cytokine receptors [60,61,62,63]. In B lymphocytes, TRAF3 directly binds to two receptors pivotal for B cell biology, the BAFF receptor (BAFF-R) and CD40, which are essential for B cell survival and activation [64,65]. Deletions and inactivating mutations of the *TRAF3* gene are some of the most frequent genetic alterations in a variety of human B cell malignancies, including MM, gastric and splenic marginal zone lymphoma (MZL), DLBCL, B-CLL, HL and Waldenstrom’s macroglobulinemia (WM) [63,66]. Corroborating the human evidence, specific deletion of the *Traf3* gene in B lymphocytes (B-*Traf3*^−/−^) causes remarkably prolonged survival of mature B cells independent of BAFF, which eventually leads to spontaneous development of splenic MZL and B1 lymphoma in mice, demonstrating that TRAF3 is a tumor suppressor in B cells [66,67,68,69]. In an effort to elucidate the metabolic basis of TRAF3-mediated regulation of mature B cell survival, we performed metabolomic, lipidomic and transcriptomic profiling to compare the metabolism of resting splenic B cells purified from young adult B-*Traf3*^−/−^ and littermate control mice. We found that multiple metabolites, lipids and enzymes regulated by TRAF3 in B cells are clustered in the choline metabolic pathway [59]. Using stable isotope labeling, we showed that the biosynthesis of P-Cho and PC species (32:2 and 34:3) is increased in *Traf3*^−/−^ B cells, which is mediated by the up-regulated expression of Chkα [59]. We also demonstrated that *CHKA* is consistently up-regulated in human MM cells that contain *TRAF3* deletions or inactivating mutations [59]. Furthermore, the reconstitution of TRAF3 in these MM cells results in decreased CHKα protein levels, reduced biosynthesis of P-Cho and PC species (32:2 and 34:3), and increased apoptosis [59]. Taken together, our findings revealed that loss of the tumor suppressor TRAF3 is a common oncogenic alteration leading to up-regulated CHKα expression and elevated choline metabolism in human and mouse B cell malignancies.

Given the increased transcript levels of *Chka* in TRAF3-deficient mouse and human B cells, we attempted to identify the transcription factor that drives *Chka* up-regulation in B cells. In light of our previous evidence that constitutive NF-κB2 activation is a major oncogenic pathway downstream of *Traf3* deficiency [63,66,67,69], we scanned the promoter regions of the mouse *Chka* and human *CHKA* genes for the presence of potential NF-κB binding sites. We found multiple consensus NF-κB binding sites in both mouse and human promoter regions. We next searched the Gene Expression Omnibus (GEO) datasets for *Chka* expression levels in mouse B cells genetically deficient in different subunits of NF-κB2 and NF-κB1 in comparison to wild type (WT) B cells. Interestingly, *Chka* expression levels are not down-regulated in *Nfkb2*^-/-^ (GSE75762 and GSE75762), *Rela*^−/−^ (GSE58972), *Relb*^−/−^, *cRel*^−/−^ or *Relb*^−/−^*cRel*^−/−^ (GSE62559) B cells as compared to WT B cells in the absence or presence of stimulation with BAFF, CD40 or CD40 plus anti-IgM [70,71,72]. However, it is evident that *Chka* expression is induced by BAFF or CD40 in mouse splenic B cells (GSE75762, GSE75762 and GSE62559). These data suggest that *Chka* up-regulation may not be directly driven by NF-κB2 or NF-κB1 in B cells, but it is a downstream event of the BAFF/CD40-TRAF3 signaling axis. We further compared the expression levels of transcription factors that are known to directly regulate *Chka* expression in other cell types, including AP1, c/EBPβ, SP1 and HIF1α [10,73,74,75,76], between *Traf3*^−/−^ and WT B cells. However, we did not detect any differences in the expression levels of these transcription factors between the two genotypes of B cells (Gokhale and Xie, unpublished data). In summary, the above data suggest that the BAFF/CD40-TRAF3 signaling axis induces *Chka* up-regulation in B cells via an indirect yet unknown mechanism that needs to be elucidated in future studies.

Despite the unclear transcriptional mechanism, the functional importance of CHKα up-regulation has been demonstrated both in vitro and in vivo. We showed that treatment with CHKα inhibitor RSM932A or MN58B potently inhibits the survival of both mouse and human TRAF3-deficient malignant B cells in vitro [59]. In vivo administration of RSM932A or MN58B substantially decreases the spleen size, partially reduces the percentage and drastically decreases the numbers of splenic B cells in B-*Traf3*^−/−^ mice but not in littermate control mice, suggesting that elevated Chkα-mediated P-Cho and PC biosynthesis contributes to the prolonged survival of *Traf3*^−/−^ B cells in vivo [59]. Furthermore, therapies that effectively control tumor progression such as radiotherapy and chemotherapy (rituximab, CHOP, R-CHOP or AZD3965) also dramatically reduce choline metabolism and decrease CHKα activity in patients, cell lines and animal models of B cell malignancies [21,27,30,40,41,42,43,77]. These findings obtained from B cell malignancies corroborate the evidence that inhibition of choline metabolism by CHKα inhibitors or genetic silencing of CHKα by siRNA has therapeutic effects in solid tumor models [1,2,3,4,5,6,15]. Our preclinical evidence of RSM932A and MN58B in TRAF3-deficient mouse B lymphoma and human MM models provides a rational basis for future clinical implementation of CHKα inhibitors or genetic targeting of CHKα as treatments for B cell malignancies.

Notably, we detected the up-regulation of *CHKA* in several TRAF3-sufficient mouse B lymphoma and human BL cell lines [59], suggesting that CHKα overexpression can also be induced via TRAF3-independent oncogenic pathways in B cell malignancies. In this context, it would be interesting to investigate key oncogenic pathways that are known to cause CHKα overexpression in solid tumors, such as PI3K-mTOR-Akt, RAS-RAF-MAPK-AP1 and HDAC1-mediated epigenetic alterations, in B cell malignancies [1,9,11,17]. For example, the PI3K-mTOR-Akt axis is often aberrantly activated through a variety of anomalies, such as gene amplification or activating mutations of PI3K isoforms, abnormal B cell receptor (BCR)-BTK or Toll-like receptor 4 (TLR4)-MyD88 signaling, or loss of PTEN in B cell malignancies [78,79,80]. Activating mutations of the RAS-RAF axis are also well-documented in human B cell malignancies [81,82]. However, direct evidence of CHKα overexpression driven by oncogenic alterations of the PI3K-mTOR-Akt or RAS-RAF pathway in malignant B cells is still lacking and awaits investigation. A recent study by Pera et al. reported that an epigenetic drug and inhibitor of lysine deacetylase (KDAC), panobinostat, elevates choline metabolism and induces CHKα up-regulation in DLBCL cells, leading to an increased reliance on CHKα for survival in these cells [83]. Consequently, treatment with the CHKα inhibitor CK37 is effective at sensitizing DLBCL cells to panobinostat in vitro [83]. Combined administration of panobinostat and CK37 significantly suppresses tumor growth, but each drug alone does not show any effect in mouse xenograft models of patient-derived chemotherapy-refractory DLBCL [83]. Therefore, a better understanding of additional oncogenic pathways that cause CHKα overexpression in B cell malignancies will provide novel insights to expand potential combination therapies exploiting CHKα inhibitors and available drugs that target relevant oncogenic pathways.

**Figure 2:**
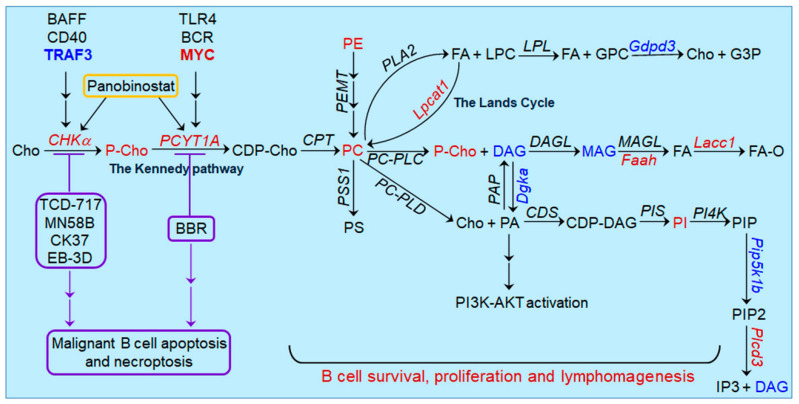
Oncogenic pathways upstream and downstream of aberrant choline metabolism discovered in B cell malignancies. Pathway schematics depict the anabolic and catabolic pathways of PC. Enzymes are denoted in Italic font in the schematics. Metabolites, lipids and metabolic enzymes that are dysregulated in malignant B cells are indicated in red (for up-regulated) or blue (for down-regulated). Available evidence shows that overexpression of two key enzymes, CHKα and PCYT1A (also known as CCT), are overexpressed in malignant B cells and mediate increased PC biosynthesis. Loss of the tumor suppressor TRAF3 and elevation of the oncoprotein MYC are common oncogenic alterations that induce the overexpression of CHKα and PCYT1A in B cell malignancies, respectively. In addition, BAFF or CD40 stimulation induces the expression of CHKα, while TLR4 or BCR engagement induces the expression of PCYT1A in normal B lymphocytes. Expression of both CHKα and PCYT1A is also induced by the KDAC inhibitor panobinostat in DLBCL cells. Several catabolic products of PC such as DAG, PA, LPC and arachidonic acid (a fatty acid) can act as lipid second messengers to induce the activation of downstream signaling pathways. Specifically, PA has been shown to activate the PI3K-AKT pathway in DLBCL. Therapeutic targeting of CHKα by pharmacological inhibitors (TCD-717, MN58B, CK37 or EB-3D) or PCYT1A by the lipid-lowering alkaloid BBR induces apoptosis and necroptosis of malignant B cells both in vitro and in vivo, indicating a causal role for CHKα or PCYT1A overexpression in B lymphomagenesis.

Besides *Chka*, we detected altered expression levels of several other enzymes of the choline metabolic pathway, including up-regulated expression of *Lpcat1*, *Faah* and *Plcd3* as well as down-regulated expression of *Dgka*, *Gdpd3* and *Pip5k1b* in *Traf3*^−/−^ B cells [59] (Figure 2). In line with our finding, up-regulation of *Lpcat1* and *Faah* or down-regulation of *Dgka* and *Pip5k1b* has been reported in other human cancers [84,85,86,87,88,89]. Among these, Lpcat1, a key enzyme of the Lands Cycle (an alternative pathway of PC synthesis) that converts LPC to PC, has been shown to play important roles in cancer pathogenesis and progression [84,85,86]. Thus, dysregulation of these enzymes may also contribute to the elevated levels of PC, phosphatidylethanolamine (PE) and phosphatidylinositol (PI) as well as the decreased levels of diacylglycerol (DAG) and monoacylglycerol (MAG) observed in *Traf3*^−/−^ B cells (Figure 2). Of particular interest, MYC, an oncoprotein frequently elevated or activated in many B cell malignancies, acts as a positive regulator of choline metabolism in DLBCL by inducing the transcriptional up-regulation of phosphate cytidylyltransferase 1 choline-α (PCYT1A) (also known as CTP:phosphocholine cytidylyltransferase-α, CCT), the rate-limiting enzyme of the Kennedy pathway of PC biosynthesis [48] (Figure 2). PCYT1A is also up-regulated by lipopolysaccharide (LPS, a ligand of TLR4) or anti-BCR in normal B lymphocytes and plays crucial roles in normal B cell proliferation, activation, GC reaction, class switch recombination and antibody secretion [32,33,34,35]. Moreover, *PCYT1A* transcription is induced by the KDAC inhibitor panobinostat in DLBCL cells [83]. MYC-driven PCYT1A up-regulation and aberrant choline metabolism impede a mitophagy-dependent necroptosis in DLBCL cells [48]. PCYT1A overexpression correlates with the prognostic index in DLBCL patients and can be targeted by the lipid-lowering alkaloid berberine (BBR), which exhibits an anti-lymphoma activity both in vitro and in vivo [48]. These findings suggest that PCYT1A is another regulatory node of choline metabolism and PC biosynthesis in B cell malignancies (Figure 2). In summary, more functional and mechanistic studies of key metabolic enzymes controlling the anabolic and catabolic pathways of PC in B cell malignancies are required to improve our understanding of lymphomagenesis as well as the treatment strategies for these diseases.

## 4. Up-Regulated Expression of CHKα and Choline Metabolism in T Cell Malignancies

Similar to that described for B cell malignancies, metabolomic analyses of serum samples by Xiong et al. showed that serum levels of choline and LPC are significantly and consistently decreased in patients with a variety of T cell lymphomas (TCL), including peripheral TCL (PTCL, not otherwise specified), cutaneous TCL (CTCL), T-lymphoblastic lymphoma (T-LBL), anaplastic large cell lymphoma (ALCL), angioimmunoblastic TCL (AITL) and natural killer/TCL [90]. The authors found that up-regulation of *CHKA* is the major cause of elevated choline metabolism in various TCL cells, resulting in decreased serum choline and LPC levels in the patients [90]. Another study by Mariotto et al. reported that both CHKα and CHKβ protein levels are up-regulated in malignant T cells of pediatric patients with T cell acute lymphoblastic leukemia (T-ALL) and a panel of T-ALL cell lines [91]. Mariotto et al. also noticed that CHKβ appears to be the predominant isoform expressed in normal T lymphocytes [91]. Paradoxically, *CHKB* expression is significantly down-regulated in patients with ALCL, PTCL and acute adult T cell leukemia/lymphoma (A-TCL/L) according to the datasets available at Oncomine [54,92,93]. Taken together, although the change of CHKβ expression in T cell malignancies needs to be further clarified, CHKα expression is consistently up-regulated in malignant T cells of patients with various TCL and T-ALL.

The causal role and functional importance of CHKα overexpression and elevated choline metabolism in aberrant survival and proliferation of malignant T cells have been demonstrated by CHKα inhibitors or genetic silencing of *CHKA* in malignant T cells [90,91]. Treatment with the CHKα inhibitor CK37 or transfection with SiRNA of *CHKA* inhibits the proliferation of the T cell line Jurkat cells via inducing apoptosis and necroptosis [90]. Inhibition of CHKα by MN58B also disrupts the balance of lipid metabolites, resulting in an increase in cellular ceramides to induce apoptosis in Jurkat cells [94]. In vivo administration of CK37 effectively suppresses tumor growth and restores serum levels of LPC in a mouse xenograft model with subcutaneous injection of Jurkat cells [90]. Similarly, the CHKα inhibitor EB-3D, RSM932A or MN58B displays potent anti-proliferative activity against a wide cohort of T-leukemic cell lines as well as primary T-ALL cells collected from pediatric patients at diagnosis via inducing apoptosis [91]. Biochemical evidence showed that EB-3D dose-dependently reduces P-Cho, PC and tCho in the T cell leukemia cell line CCRF-CEM cells, confirming its action on CHKα-mediated choline metabolism [91]. Interestingly, EB-3D enhances the sensitivity of T-ALL cells to drugs used in standard therapeutic protocols such as dexamethasone (DEX) and l-asparaginase (l-ASP). EB-3D synergizes with both DEX and l-ASP at inhibiting the proliferation of T-ALL cells by decreasing the GI50 of the two drugs and by increasing the percentage of apoptotic cells compared to the single treatments [91]. Thus, malignant T cells exhibit a reliance on CHKα overexpression for survival, proliferation and tumorigenicity, indicating an oncogenic role of CHKα overexpression and the resultant elevated choline metabolism in TCL and T-ALL. However, information regarding how CHKα expression is up-regulated in T cell malignancies is still lacking and needs to be elucidated in future studies.

In light of the evidence that HDAC inhibitors induce CHKα up-regulation in human DLBCL and solid tumors [17,83], it would be interesting to test if CHKα overexpression is caused by epigenetic alterations in various T cell malignancies and if HDAC inhibitors induce CHKα up-regulation in malignant T cells. This is particularly important and clinically relevant given that HDAC inhibitors have been approved by the U.S. Food and Drug Administration (FDA) for the treatment of CTCL and PTCL [95].

## 5. Oncogenic Pathways Downstream of CHKα Overexpression in Lymphoid Malignancies

Emerging evidence revealed that CHKα overexpression induces the activation of downstream oncogenic pathways such as the PI3K-mTOR-AKT, Ras-ERK/AKT-MYC and AMPK-mTORC1 axes in B cell and T cell malignancies, analogous to that observed in solid tumors [3,15]. CHKα overexpression mediates increased biosynthesis of PC. Catabolic metabolism of PC generates lipid second messengers, including DAG, phosphatidic acid (PA), LPC and arachidonic acid, which in turn can activate various downstream signaling pathways [96,97,98,99]. In DLBCL cells, the KDAC inhibitor panobinostat induces CHKα up-regulation, which is associated with the activation of the PI3K-AKT pathway [83]. Genetic silencing of CHKα or treatment with the CHKα inhibitor CK37 abrogates the apoptosis-inducing effects of the PI3K inhibitor AZD8186 in DLBCL cells, indicating a dependence of PI3K activation on CHKα up-regulation [83]. Interestingly, exogenous addition of PA rescues DLBCL cells from CK37-induced apoptosis and restores the activation of PI3K-AKT, suggesting that PA is a second messenger linking CHKα up-regulation to PI3K activation in DLBCL [83]. For T cell malignancies, pharmacological inhibition or genetic silencing of CHKα significantly decreases the activity of Ras-GTP, the active form of Ras protein, reduces the levels of ERK and AKT phosphorylation, and down-regulates the expression of MYC oncoprotein both in cultured TCL cells in vitro and in a mouse model of TCL xenografts in vivo [90]. Correlation between CHKα up-regulation and increased levels of p-ERK, p-AKT and MYC is also detected in tissue samples of TCL patients [90]. Furthermore, pharmacological inhibition of CHKα induces rapid activation and phosphorylation of the metabolic stress sensor AMP-activated protein kinase (AMPK), which in turn represses the mTORC1 pathway in T-ALL cells [91]. However, it is unclear whether a feed-forward amplification loop formed by the reciprocal interaction between CHKα overexpression and other oncogenic pathways that have been elucidated in solid tumors [1,3,15] exists in malignant B cells and T cells. In addition, a non-catalytic scaffolding function of CHKα has been shown to promote cancer pathogenesis by linking growth receptor signaling to lipid biogenesis in solid tumors [3,15], but it remains unknown whether this type of signaling is relevant to lymphomagenesis.

## 6. Concluding Remarks and Perspectives

Initial MRS imaging studies showed that abnormal choline metabolism is detectable in patients and animal models with NHL and CNSL. More recently, PET/CT imaging with choline tracers has become a useful tool for staging and assessment of therapeutic response in patients with lymphoid malignancies, demonstrating much higher sensitivity and specificity for MM and CNSL than the widely used glucose tracer. Metabolomic analyses of serum or plasma samples of patients and animal models with lymphoid malignancies also reveal evidence of choline metabolic alterations, including decreased serum or plasma levels of choline, PC, LPC and GPC. Thus, both the imaging and metabolomic data indicate that increased choline uptake and elevated choline kinase activity are ubiquitously present in lymphoid malignancies. However, genetic alterations of *CHKA* and *CHKB* are rare events in human lymphoid malignancies according to the TCGA datasets. Interestingly, CHKα overexpression is consistently observed in both patients and animal models of various B cell and T cell malignancies. Targeting of CHKα by pharmacological inhibitors or genetic silencing has demonstrated anti-tumor efficacy in preclinical models of lymphoid malignancies both *in vitro* and *in vivo*. Furthermore, preclinical evidence also shows the potential of CHKα inhibitors in combination therapies when applied together with other commonly used clinical drugs of lymphoid malignancies such as HDAC inhibitors, DEX and l-ASP. Such preclinical evidence lays the foundation for future clinical implementation of CHKα inhibitors as treatments for lymphoid malignancies. However, it should be noted that the CHKα inhibitor TCD-717 (also named RSM932A) has completed the first Phase I clinical trial in patients with advanced solid tumors (NCT01215864, https://www.clinicaltrials.gov/ (accessed on 8 June 2021)) in 2014, but no results of this trial have been published or uploaded to ClinicalTrials.gov to date. Given this extended lag of result publication, it is likely that the efficacy or safety profile of TCD-717 is not as promising as initially envisioned. In such cases, future clinical trials of TCD-717 or other CHKα inhibitors need to consider altering the dosage or administration schedule and focusing on combination therapies with other drugs rather than monotherapy to improve the efficacy of CHKα inhibitors while minimizing the toxicity. Alternatively, more effective and better-tolerated CHKα inhibitors should be developed for clinical use in the treatment of both solid tumors and lymphoid malignancies.

Although the value of CHKα overexpression as a diagnostic biomarker and therapeutic target is well recognized, how CHKα overexpression is induced in lymphoid malignancies has just begun to be unraveled. We recently found that loss of the tumor suppressor TRAF3 is a common oncogenic alteration leading to up-regulated CHKα expression and elevated choline metabolism in human and mouse B cell malignancies. *Chka* expression is also induced by BAFF or CD40 in mouse splenic B cells, suggesting that *Chka* up-regulation is a downstream event of the BAFF/CD40-TRAF3 signaling axis in B cells. However, we also detected up-regulation of *CHKA* in TRAF3-sufficient mouse B lymphoma and human BL cell lines, indicating that CHKα overexpression can be induced via TRAF3-independent oncogenic pathways in B cell malignancies as well. In this regard, several key oncogenic pathways that are known to cause CHKα overexpression in solid tumors such as PI3K-mTOR-Akt and RAS-RAF-MAPK-AP1 are often aberrantly activated in lymphoid malignancies. Direct evidence of CHKα overexpression driven by oncogenic alterations of the PI3K-mTOR-Akt or Ras-Raf pathway in malignant B cells or T cells is still lacking and thus requires further investigation. A better understanding of additional oncogenic pathways that deregulate CHKα expression in lymphoid malignancies will provide novel insights to expand potential combination therapies exploiting CHKα inhibitors and available drugs that target relevant oncogenic pathways.

Besides *Chka*, altered expression levels of several other enzymes of the choline metabolic pathways are detected in *Traf3*^−/−^ B cells, including *Lpcat1*, *Faah*, *Dgka* and *Pip5k1b*. Dysregulation of these enzymes has been reported in other human cancers and may also contribute to elevated choline metabolism. Interestingly, the up-regulation of PCYT1A, the rate-limiting enzyme of the Kennedy pathway of PC biosynthesis, is induced by the oncoprotein MYC and mediates elevated choline metabolism in human DLBCL, suggesting that PCYT1A is another therapeutic target of choline metabolism. However, PCYT1A is also up-regulated by LPS and anti-BCR in normal B lymphocytes and plays crucial roles in normal B cell proliferation, activation, GC reaction, class switch recombination and antibody secretion. In line with this, the specificity of choline tracers is not absolute for lymphoid malignancies, as [^11^C]-choline or [^18^F]-fluorocholine is also accumulated in tissues with sterile inflammation and bacterial/viral infections. In this context, an important area for future research is to elucidate whether therapeutic targeting of choline metabolism by pharmacological inhibitors specific for CHKα, PCYT1A or other enzymes affects B cell and T cell immune responses during bacterial and viral infections. In summary, additional functional and mechanistic studies of key metabolic enzymes controlling the anabolic and catabolic pathways of PC in both immune responses and lymphoid malignancies are needed to improve our understanding of adaptive immunity and lymphomagenesis as well as the diagnostic and therapeutic strategies for lymphoid malignancies.

## Data Availability

Not applicable.
